# A journal article reporting standard working group in sport and exercise science: a call to action

**DOI:** 10.1007/s00421-026-06242-5

**Published:** 2026-04-21

**Authors:** Konstantin Warneke, Thomas Gronwald, Filipe Manuel Clemente, Renato Andrade, José Afonso

**Affiliations:** 1 Institute of Sustainability Psychology, Leuphana University Lüneburg, Lüneburg, Germany; 2 Institute of Interdisciplinary Exercise Science and Sports Medicine, MSH Medical School Hamburg, Hamburg, Germany; 3 Applied Research Institute (i2A), Polytechnic University of Coimbra, Coimbra, 3045- 093 Portugal; 4 Department of Biomechanics and Sport Engineering, Gdansk University of Physical Education and Sport, Gdańsk, 80-336 Poland; 5 Sport Physical Activity and Health Research & Innovation Center, Polytechnic University of Coimbra, Coimbra, 3045-093 Portugal; 6 Clínica Espregueira - FIFA Medical Centre of Excellence, Porto, Portugal; 7 Dom Henrique Research Centre, Porto, Portugal; 8 Porto Biomechanics Laboratory (LABIOMEP), Faculty of Sports, University of Porto, Porto, Portugal; 9 Centre of Research, Education, Innovation, and Intervention in Sport (CIFI2D), Faculty of Sport, University of Porto, Porto, Portugal

## Why we need journal article reporting standards in sport and exercise science

Evidence-based practice is the gold-standard in general health and performance training, with sport scientists working to bridge the gap between science and practice. To understand and communicate scientific results and review whether study protocols and data collection were performed under standardized conditions to produce trustworthy results, journal articles must present the study methodology and results in the most comprehensive way possible, containing crucial information about the evaluation process and how results were obtained, preferably preceded by ethical approval (when applicable) and protocol pre-registration (Afonso et al. 2026). In a field with a rapidly expanding literature, this is not merely a matter of editorial style, since physical activity and exercise studies have shown suboptimal adherence to recommended reporting and methodological practices (Oliveira et al. 2021). Systematic reviews repeatedly concluded that most exercise interventions (72%) were insufficiently described for replication and translation into practice (Hansford et al. 2022; Murphy et al. 2025). Consequently, the current landscape reveals substantial heterogeneity in how quantitative studies are designed, analyzed, and reported. When systematic reviews are performed about sport and exercise science topics, the certainty of evidence is typically judged as low to very low, mostly due to the initial low starting level of a body of evidence (Murphy et al. 2025).

## The EQUATOR list

The Equator Network contains hundreds of guidelines on how to structure different types of articles and where specific information should be reported within the article (The EQUATOR Network 2026). Randomized controlled studies should follow the CONSORT 2025 guidelines that endorse reporting standards about study design, data collection, and how the relevant information must be reported in their publication (Hopewell et al. 2025). Given the plurality of study designs and the specificities of each research field, there are numerous specific guidelines and extensions that adjust the reporting recommendations to specific fields and topics.

Remarkably, out of 699 guidelines (March 2026) listed in the EQUATOR Network, only the Consensus on Exercise Reporting Template (CERT) (Slade et al. 2016) is suitable for sport and exercise science intervention studies. CERT should be used as complement to CONSORT and American Psychological Association (APA) Journal Article Reporting Standards (JARS) (Appelbaum et al. 2018), as it is focused on how exercise routines should be described with (e.g., materials, provider, delivery, location, dosage, tailoring, and compliance)(Slade et al. 2016). Moreover, some features of general guidelines (e.g. CONSORT 2025) can hardly be implemented when researching exercise programs, such as blinding of participant and intervention provider (although it may be possible, in certain contexts, to blind them to the research goals). What is lacking is a thorough, sport and exercise science-specific guide on how to collect data and produce a comprehensive journal article that reports relevant and highly-specific information, which is imperative for ensuring optimal data quality, contextualization, interpretation, and comparability of the findings. This is needed to improve comprehensiveness and homogeneity in results reporting and implement quality standards that authors must adhere to when collecting data and publish in high-quality scientific journals.

Reporting quality should not be conflated with methodological quality or risk of bias. Better reporting does not guarantee valid inference, but inadequate reporting prevents readers from judging validity at all. For that reason, a minimum set of relevant items for quantitative studies must be developed. These can adopt aspects from other guidelines, e.g. randomization procedures, allocation concealment, blinding, a priori sample-size justification, handling of multiple testing and data imputation, adverse events, and transparent reporting of intervention fidelity and adherence beyond simple session attendance. Nevertheless, there are numerous sport and exercise science specific aspects that must be identified and addressed. For instance, significant habituation effects conflate internal data validity, especially under unfamiliar testing conditions (Ploutz-Snyder and Giamis 2001; Ritti-Dias et al. 2011). The research landscape is heterogeneous in how to quantify measurement errors, reliability, validity and objectivity, if it was reported at all. While most authors quantify reliability via Intraclass Correlation Coefficients, earlier initiatives requested the addition of agreement analyses (Atkinson and Nevill 1998), which is still insufficiently implemented in the field of sport and exercise science research, as well as sport psychology. Related guidelines raised awareness on criteria such as internal consistency and the interaction with test specificity (Appelbaum et al. 2018), but consensus is missing in sport and exercise science, causing confusion on interchangeability of test scores. For instance, Warneke et al. (2023) highlighted that, despite strong correlations, dynamic and isometric strength tests cannot be considered interchangeable, particularly given differing testing objectives – a typical assumption and recommendation in sport and exercise science to make tests more practically applicable (McGuigan and Winchester 2008; McGuigan et al. 2010).

## Journal article reporting standards working group

Implementation of APA standards was initiated in 2006 by appointing a Working Group on Journal Article Reporting Standards by the Publications and Communications Board of the APA (APA Publications and Communications Board Working Group on Journal Article Reporting Standards 2008; Appelbaum et al. 2018). The CONSORT guidelines are regularly updated by an expert panel (e.g., CONSORT 2025). However, a Publications and Communications Board as well as a JARS working group do not exist in sport and exercise science, neither are there any globally accepted and implemented guidelines hosted by international associations (e.g., European College of Sport Science, American College of Sports Medicine). This leads to ambivalent scientific results reporting quality in sport science that not only downgrades research synthesis in reviews and meta-analysis but also slows down science advancements and transfer to applied fields. An insufficiently detailed reporting will likely result in poorly implemented or misguided interventions.

To address this gap, this comment was written to raise awareness for these limitations in our field and a call to action for forming a JARS working group that regularly updates guidelines considering topic specific requirements in orientation on existing reporting standards and related fields provided via the EQUATOR Network. Given the large heterogeneity in our field, medical research might act as a role model with a core standard guideline (e.g., CONSORT 2025), supplemented with subfield-specific extensions to cover specificities of the respective fields (e.g., resistance training and endurance training might differ within exercise science, while also no sport psychology specific guideline exist, which will probably differ from those in exercise science). This process should be supported by high-quality journals and their editorial teams by implementing reporting standards as a baseline for rigorous publications. One way to achieve this is by providing a checklist to guide authors and ensure that critical information is consistently and transparently reported, while informing readers that studies which adhere to these guidelines meet the high standards of reporting quality. This checklist could orientate on existing guidelines and adjusted for sport and exercise science (e.g., how to perform and report pre-study registration, accounting for reliability, validity and objectivity, or testing specificity, among others). By providing clear expectations, such standards would support authors during study planning and manuscript preparation, assist peer-reviewers and editors in completeness and quality appraisal, and enhance comparability across studies. Implementing such standards will also improve the data extraction process for systematic reviews which is important to avoid calculation errors and misleading conclusions.

Developing such guidelines requires collective expertise and broad representation across sub-disciplines, methodological traditions, and applied contexts. A structured consensus process offers a transparent and inclusive pathway forward. In this regard, the Delphi method provides a well-established framework for systematically gathering expert opinion through iterative survey rounds, controlled feedback, and predefined consensus criteria. By engaging researchers, practitioners, statisticians, journal editors, and early-career scholars, a Delphi-based initiative can identify core reporting domains and define priority items that reflect the real needs of the field.

We therefore invite members of the sport and exercise science community to actively participate in an upcoming Delphi consensus process as a first initiative of a JARS working group for quantitative research guidelines on sport and exercise science. The implementation of reporting standards will not solve all problems in the field but is crucial to transparently communicate scientific findings into practice and enhance trustworthiness and credibility of our field and support the translation of scientific findings into meaningful practice. To achieve this goal, your expertise irrespective of the exact field is essential. High-quality standards as a methodological foundation for future research can only emerge from broad engagement and critical dialogue. We call upon the community to join this effort and to collectively define what high-quality quantitative research reporting should look like in sport and exercise science with a subsequent discussion about subfield extensions by forming interdisciplinary and interprofessional expert panels for these individual research areas. Together, we can ensure that the growth of our field is guided not only by productivity, but by rigor, transparency, and lasting impact.

## Data Availability

Not applicable
